# Identifying clinically relevant subgroups of patients with knee pain flares for ibuprofen treatment: a secondary analysis

**DOI:** 10.1007/s10067-025-07539-0

**Published:** 2025-06-25

**Authors:** R. U. Sharma, J. Runhaar, P. K. Bos, D. M. J. Dorleijn, P. J. E. Bindels, S. M. A. Bierma-Zeinstra

**Affiliations:** 1 Department of General Practice, Erasmus MC University Medical Center Rotterdam, Rotterdam, The Netherlands; 2 Department of Orthopedics & Sports Medicine, Erasmus MC University Medical Center Rotterdam, Rotterdam, The Netherlands; 3 Department of Orthopedics and Traumatology, University Hospital Ghent, Ghent, Belgium

**Keywords:** Ibuprofen, Knee joint, Knee pain, Morning stiffness, Osteoarthritis, Swelling

## Abstract

**Objective:**

We aimed to evaluate whether subgroups with more severe inflammatory symptoms during a knee pain flare benefited more from a high dose ibuprofen treatment than subgroups with less severe inflammatory symptoms.

**Methods:**

This secondary analysis included adults with ≥ 1 flares of knee pain in the last year, who experienced a new episode within 24 h and randomized into two treatment groups of daily ibuprofen 1200 mg or 2400 mg for 5 days. A multilevel regression analysis was used to assess interaction effects between intervention groups and pre-defined subgroups, based on osteoarthritis related symptoms (severity of morning stiffness, swelling, and pain). The primary outcome was the difference in treatment effect between subgroups on pain severity (0–10 on the numeric rating scale (NRS)) after 5 days. Differences in treatment effect between subgroups after 3 days (NRS) and 5 days (Western Ontario and McMaster Universities Osteoarthritis (WOMAC) scale) were secondary outcomes.

**Results:**

Participants (*N* = 308) had a mean age of 52.4 ± 12.9 (SD) years with 41% female subjects. No significant interaction was found between the pre-defined subgroups and intervention groups on pain severity after day 5 (all *p*-values ≥ 0.28) or on the secondary outcomes (all *p*-values ≥ 0.38). Given the potential lack of power, the absolute and adjusted mean differences between treatment arms were compared for each subgroup; none of the differences reached clinical significance.

**Conclusion:**

Between subgroups with more and less severe inflammatory symptoms during knee pain flares, no significant nor clinical benefit was found from a higher dose of ibuprofen compared to a lower dose.

**Table:** 

**Keypoints** • *Despite the overall superiority of the higher dose, patients with severe inflammatory knee symptoms do not benefit more from an anti-inflammatory dose of ibuprofen than patients with less severe symptoms.* • *A higher dose of ibuprofen is not indicated for patients with severe inflammatory knee symptoms.* • *Given the heterogeneity among patients with knee osteoarthritis, potential subgroups should be explored in future research.*

**Supplementary Information:**

The online version contains supplementary material available at 10.1007/s10067-025-07539-0

## Introduction

Knee pain is a frequently reported complaint and affects up to 25% of adults above the age of 55 years [1, 2]. Knee pain limits function and mobility, which in its turn reduces quality of life [3]. In the natural course of early and advanced stages of osteoarthritis (OA), flares or flare-ups, described as intermittent, disabling pain episodes, are not uncommon [4–6]. There is strong evidence to support the hypothesis that joint inflammation is associated with structural and clinical progression of OA. This includes worsening of synovitis and is associated with frequent knee pain and fluctuation in pain intensity, which are elements of flares [7–9]. Episodic flares are triggered by a wide range of activity-related, psychosocial, and environmental factors, leading to worsening of the pain and functional impairment [6, 10, 11]. Within-person changes in joint effusion are associated with fluctuations in knee pain severity [9]. During an episodic flare, patients may present with swelling and the physician can assess effusion of the knee joint, implying an inflammatory component with changes in synovial fluid [3].

Due to a potential underlying inflammation process, prompt (short-term) suppression of inflammation could slowdown joint destruction and symptom progression due to OA. For acute episodes of knee pain in OA, non-steroidal anti-inflammatory drugs (NSAIDs), in particular ibuprofen, are the most commonly prescribed therapy in practice and conditionally recommended by clinical practice guidelines [12–14]. Ibuprofen is well-tolerated, contains a well-established safety profile [3], and is widely used as an analgesic, anti-inflammatory, and antipyretic agent [15, 16].

Unfortunately, contra-indications and a wide variety of adverse reactions (e.g., gastrointestinal symptoms or changes in kidney function) of NSAIDs limit their use. Since a higher dose of NSAID is associated with more adverse events, the dose should be limited [16, 17]. Yet, patients with elevated signs of inflammation might benefit from a higher dose of NSAIDs [15, 18, 19].

The Global Burden of Disease 2017 study encouraged targeted therapy in prevention and treatment management to specific groups with a high burden of OA [20]. However, it is known that the population of knee OA patients is heterogeneous. Also, current symptomatic treatment modalities for knee OA have low to moderate effects [21]. Given these facts, there is a general call for the evaluation of clinically relevant subgroups to allow for targeted therapy [22–25].

A randomized controlled trial (RCT), the “Flaring Arthralgia Relief Evaluation in episodic flaring knee pain” (FLARE), by Bierma-Zeinstra et al. compared the effect of 1200 mg and 2400 mg ibuprofen among patients with knee pain who reported a recent flare of symptoms [3]. This study showed a small statistical significant (effect size 0.4) dose-related benefit after five days of treatment with 2400 mg ibuprofen over 1200 mg, among individuals with a flare of knee pain. The upper limit of the confidence interval (CI) of this treatment effect reached beyond the clinically relevant margin. This overall small beneficial effect and potential clinical relevancy could be an indication for possible subgroup treatment effects.

Therefore, this secondary explorative data analysis aimed to evaluate pre-defined potential subgroups of patients who will benefit from a high dose of NSAIDs. We hypothesized that patients who have more severe self-reported morning stiffness, more severe sensation of swelling in the knee, or a higher knee pain score at baseline, compared to patients with less severe of these inflammatory symptoms, would respond better to 2400 mg ibuprofen as compared to 1200 mg ibuprofen after five days of treatment.

## Materials and methods

### Study design

This study was a pre-specified secondary analysis of the dataset of the FLARE RCT; a multicenter, 1:1 randomized, double-blind, 3-arm, non-inferiority trial comparing a 5-day course of different ibuprofen regimens [3]. The focus for this secondary analysis were the participants randomized to a 5-day treatment course with a total daily oral dose of either 1200 mg or 2400 mg ibuprofen. The first regimen consisted of one ibuprofen soft-gel capsule of 400 mg plus one placebo capsule, three times daily. The second regimen consisted of two ibuprofen soft-gel capsules of 400 mg, three times daily. The third treatment arm with 1200 mg lipid capsule of the original study was disregarded in this analysis, as it is not widely available and experimental. Day 0 was the baseline day (no medication), and day 1 was the first treatment day. The study was conducted at 27 primary care centers, located in the UK and the Netherlands. The RCT followed an intention-to-treat analysis for their main analysis, involving all 464 randomized study participants. Self-reported morning stiffness, sensation of swelling in the knee, and severity of knee pain, which are all possible indicators of inflammation, were assessed at baseline. Demographic data and baseline characteristics were recorded by the investigators by reviewing their medical healthcare records. The intervention consisted of a 5-day ibuprofen treatment course with an average patient compliance of greater than 97% in all groups. All study drugs were produced by the same manufacturer and presented as identical capsules to preserve blinding [3].

The full protocol details of the RCT can be found in the EU Clinical Trials Register (EudraCT number: 2014–004254-33) [3, 26]. All participants signed a written informed consent, and the study was approved by the National Research Ethics Service Committee East Midlands—Northampton (United Kingdom) and the Independent Review Board Nijmegen (The Netherlands).

### Study population

The population of the RCT consisted of adults aged 18–70 years, with a history of ≥ 1 knee pain flare episodes in the previous 12 months (with or without treatment), who attended a baseline evaluation within 24 h of experiencing a new knee flare pain episode. A new knee pain flare was defined as an identifiable increase in pain level (pain severity score of ≥ 5 on a 0–10 numerical rating scale (NRS)) associated with a decrease in normal daily function. The patients with a history of knee pain flares were identified by different methods, such as medical record reviewing (selecting knee pain episodes by a keyword search) and local advertisement. Baseline characteristics, such as gender and ethnicity, were self-reported from a fixed set of categories. Key exclusion criteria of the RCT were recent serious illness, diagnosis of gout, fracture, significant injury or surgery to the knee, recent intra-articular treatment or systemic corticosteroids, use of allopurinol, febuxostat or colchicine, current selective serotonin reuptake inhibitor medication, use of any oral or topical pain medication within 7 days of study baseline, and a body mass index (BMI) less than 18 or higher than 39 kg/m^2^ or a body weight less than 40 kg.

Participating patients with a value of pain severity on the NRS and Western Ontario and McMaster Universities Osteoarthritis Index (WOMAC; 3.1 Index) pain scale at least at baseline and end of treatment were included. Patients without any measurements at the end of treatment course (day 5) were excluded.

### Outcomes

The primary outcome for this secondary analysis was the difference in the change in average daily self-reported NRS pain severity score after 5 days of treatment with 2400 mg compared to 1200 mg oral ibuprofen between the predefined subgroups with more or less severe inflammatory symptoms. The secondary outcomes were the difference in the change in WOMAC pain subscale score after 5 days of treatment and the difference in the change in NRS pain severity score on day 3, both comparing effects of 2400 mg ibuprofen treatment to 1200 mg between the subgroups. The NRS pain severity was scored from 0 (no pain) to 10 (extreme pain) over the last 7 days. The WOMAC pain subscale over the last 2 days, was measured with a pre-defined 5-point scale, normalized to a 0–10 VAS range, where 0 indicates no pain. In case of no statistical significance, clinical significance was taken into consideration by comparing between-subgroup differences in treatment effect to the minimal clinically important difference (MCID).

### Data management

Anonymized data of the FLARE RCT was provided by Infirst Healthcare, manufacturers of the new lipid formulation of ibuprofen (Flarin®). Current analyzes were pre-designed and performed without input of the data owner.

First, the data was checked for available variables and missing data. Secondly, to check for reproducibility of the published data, the baseline characteristics (age, age category, gender, race, comorbidity) were assessed as well as the number of randomized patients at baseline, and the mean NRS pain severity score and WOMAC pain scores at baseline. This was done by descriptive analyzes. Missing data was assumed to be missing at random and was not imputed, as it was less than 10% of the data. The reproducibility was checked on the full analysis dataset of the primary trial.

### Subgroup definitions

Three subgroups were pre-defined based on the existing literature [22, 23] and clinical expertise, prior to testing for interactions. The domains of inflammation, associated with clinically distinct phenotypes, were used to pre-define the subgroups [22]. The subgroups were defined as more or less severe inflammatory knee symptoms at baseline based on self-reported NRS:

- Subgroup with more/less severe morning stiffness
- Subgroup with more/less severe knee swelling
- Subgroup with more/less severe pain

To determine the cut-off values of the baseline pain, morning stiffness, and swelling scores on the continuous NRS scales for each subgroup, a 30/70 ratio for dichotomization was used in the randomized population, corresponding with previous literature [27, 28]. The 30/70 distribution was a practical approach to acquire clinically relevant groups and to ensure sufficient statistical power while maintaining the validity of subgroup analyses.

### Statistical analysis

The data consisted of repeated measurements for which a mixed model linear regression was used. With this model, the potential subgroup effects were assessed by calculating the interaction between the treatment effect and each subgroup at day 3 and day 5 of treatment. The model included the following:

- dependent variable: either NRS pain (primary) or WOMAC pain score (secondary) after 5 days of treatment
- independent variable: treatment group (1200 mg or 2400 mg)
- effect modifier: subgroup indicator (more or less severe inflammatory symptoms at baseline) and
- interaction term: independent variable BY effect modifier [29].

The baseline pain score was included as a random effect in this analysis, as the variability of the baseline score between the subjects was meaningful. The treatment group, subgroup indicator, treatment center, and interaction effect were included as fixed effects. We additionally adjusted for treatment center and baseline scores of the outcome. Data from centers that contributed to less than six subjects to the primary endpoint analysis were pooled based on geographical location for analysis purposes, with small centers pooled with their nearest neighbor and pooling only occurred within each country.

The relevant effect size in this study was defined as the adjusted mean difference of the treatment interaction effect between subgroups (high/low inflammatory symptoms) and its significance level. First, the adjusted mean treatment differences (i.e., 2400 mg vs 1200 mg) within each subgroup (i.e., more vs less severe inflammatory signs) were quantified for specific time points. Secondly, the adjusted mean differences of the treatment effect between the subgroups were calculated, with their corresponding two-sided symmetric 95% CI. The interaction effect between the intervention groups and the subgroups was considered statistically significant with a *p*-value less than 0.05.

To be able to interpret the effect size of the treatment interaction in the clinic and to cater individualized therapy, a MCID was defined for this study. Patient reported outcome measures did not have a single value for the MCID, and the value varies depending on the intervention and context [30, 31]. In this study, a MCID ≥ 0.8 (on a scale of 0–10) was used to assess clinical relevance. As the scores were derived from subgroup analyses, the improvement of the MCID would be more appropriate to apply on individuals with these subgroup characteristics. All analyzes were performed using IBM SPSS Statistics V.28.0.

### Sensitivity analysis

The FLARE database included all patients with a history of knee pain flares with or without clinically defined knee OA. Taking into account that a knee pain flare is a common phenomenon in OA patients and NSAIDs are recommended as a short-term treatment during an OA flare by the clinical practice guidelines for pharmacological treatment [10, 13, 14, 32, 33], we performed a sensitivity analysis on a subset of patients with knee OA. Patients with a reported medical history of OA (i.e., diagnosed with any term indicating “knee OA” or with “OA” without indication of a specific joint, but excluding patients with a diagnosis of OA in another joint than the knee) were selected.

After creating the subset with OA patients, the methodological and statistical steps of the primary analysis to test the interaction effects, without any additional adaptation, were performed. The sensitivity analysis was performed only for the primary outcome.

## Results

**Fig. 1:**
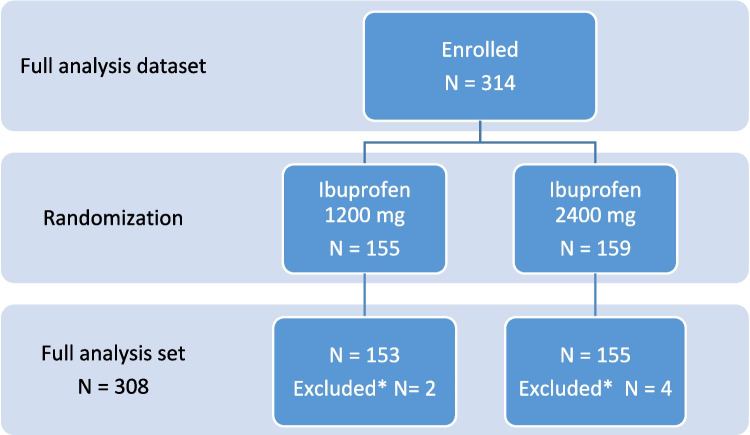
Flowchart of included patients in the secondary subgroup analysis. *Missing NRS score at day 5. *N* = number of patients; NRS = Numeric Rating Scale

The full analysis dataset for this secondary analysis consisted of 314 treated patients. After randomization, 155 patients were allocated to the treatment group with 1200 mg ibuprofen and 159 patients to the 2400 mg ibuprofen treatment group. After excluding patients with missing values, the full analysis set consisted of 308 patients (Fig. 1). However, due to missing WOMAC pain score data at follow-up, 307 patients were analyzed for this secondary outcome.

The baseline characteristics of the full analysis set of 308 patients are shown in Table 1. The cohort existed of a population with a mean age of 52.4 ± 12.9 (standard deviation (SD)) years and 41% female subjects.

**Table 1:** Baseline characteristics

	Ibuprofen 1200 mg	Ibuprofen 2400 mg	Total
N*	153	155	308
Age (mean ± SD*)	51.6 ± 13.1	53.1 ± 12.7	52.4 ± 12.9
Age-category (< 65 years) (%)	128 (84)	121 (78)	249 (81)
Sex (female subjects) (%)	63 (41)	64 (41)	127 (41)
Body mass index (mean kg/m^2^ ± SD)	28.7 ± 4.5	28.5 ± 4.5	28.6 ± 4.5
Ethnicity (White) (%)	141 (92)	148 (96)	289 (94)
Osteoarthritis (yes) (%)	65 (43)	66 (43)	131 (43)

*N = number of patients randomized to the specific group; SD = standard deviation

### Subgroup definitions

The cut-off values, based on the baseline NRS severity score, for the different subgroups are presented in supplementary Table 1.

### Treatment effects among subgroups

#### Primary outcome

**Fig. 2:**
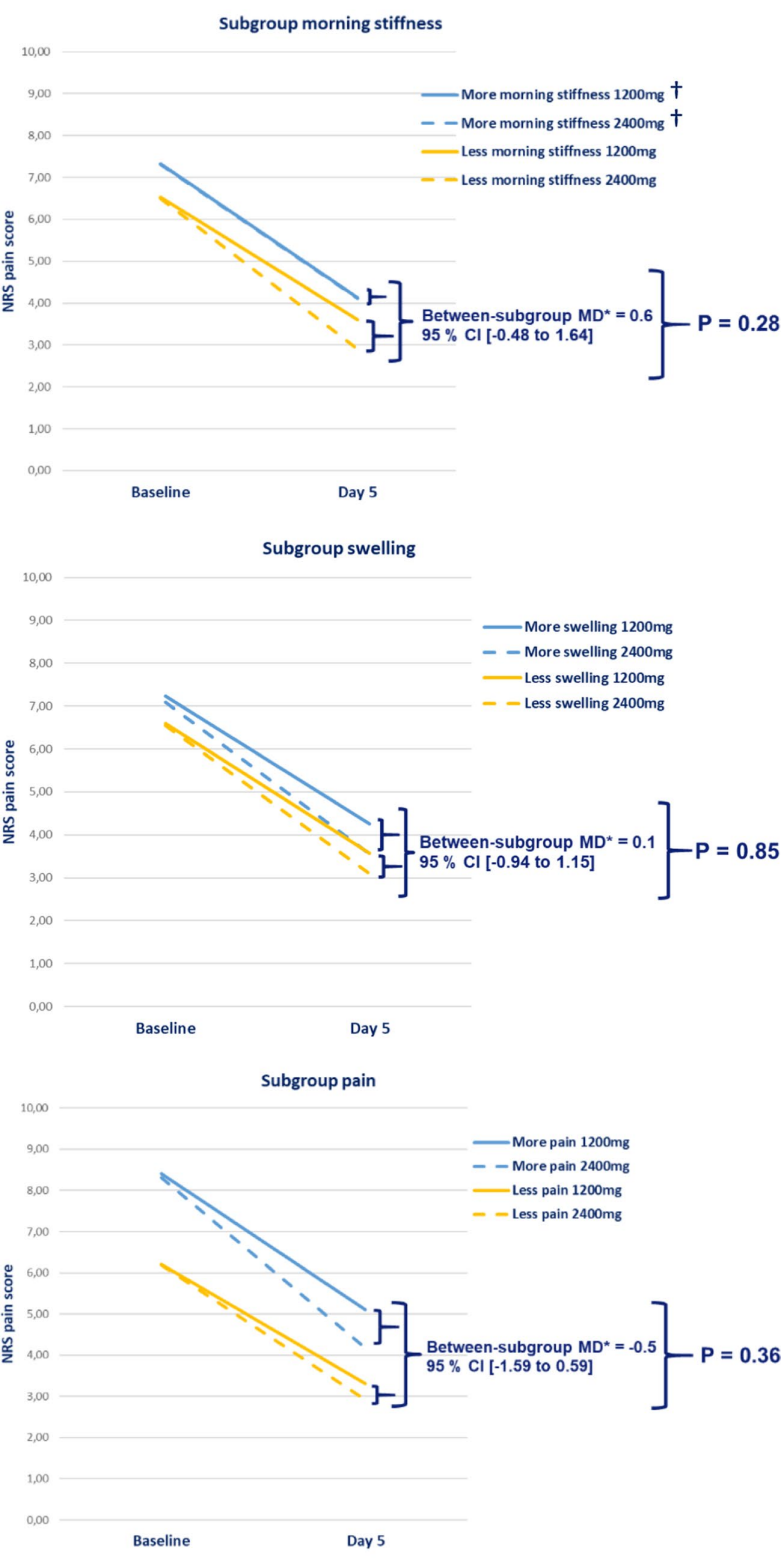
Adjusted mean between-subgroup treatment differences and corresponding significance level of the treatment effect for each subgroup on the primary outcome measure (pain severity (0–10 range)) at baseline and day 5 of treatment. †Overlap of the lines of the subgroup more morning stiffness *MD = adjusted mean difference; NRS = Numeric Rating Scale; *P* = *p*-value

No statistical significant interaction was found on the pain severity at day five between the intervention groups and morning stiffness subgroup (*p*-value = 0.28; Table 2; Fig. 2). Also, no significant interaction on pain severity at day 5 was found between the intervention groups and the subgroups swelling (*p*-value = 0.85) or pain severity (*p*-value = 0.36).

**Table 2:** Subgroup analyses. Subgroup analyses (interaction intervention × subgroup and adjusted* mean differences) for pain severity at day 5 (primary outcome), WOMAC pain at day 5 (secondary outcome), and pain severity at day 3 (secondary outcome) for *N* = 308

	Interaction *p*-value	Observed effect size	95% CI**
Morning stiffness			
Pain severity day 5	0.283	0.578	− 0.481 to 1.638
WOMAC pain day 5	0.519	0.311	− 0.638 to 1.261
Pain severity day 3	0.506	0.315	− 0.616 to 1.245
Swelling			
Pain severity day 5	0.845	0.104	− 0.943 to 1.151
WOMAC pain day 5	0.377	− 0.420	− 1.353 to 0.513
Pain severity day 3	0.795	0.122	− 0.800to 1.044
Pain			
Pain severity day 5	0.364	− 0.502	− 1.591 to 0.586
WOMAC pain day 5	0.523	− 0.318	− 1.296 to 0.660
Pain severity day 3	0.454	− 0.365	− 1.324 to 0.593

*MD = adjusted mean difference; adjusted for treatment center and baseline score of the outcome. Between-subgroup mean treatment difference is the mean difference between the subgroup with less and more severe complaints for the treatment of 2400 mg versus 1200 mg ibuprofen

**CI = confidence interval

There were no clinically relevant differences in the absolute (unadjusted), nor in the adjusted mean differences in treatment effects between the pre-defined subgroups. The adjusted mean between-subgroup treatment differences were all less than 0.58, which is below the margin of the MCID of ≥ 0.8 (Table 2).

#### Secondary outcome

For the WOMAC pain score at day 5 of treatment and NRS pain severity score at day 3 of treatment, no significant interaction effects between the intervention groups and pre-defined subgroups were found (*p*-values ≥ 0.38 and ≥ 0.45, respectively).

**Fig. 3:**
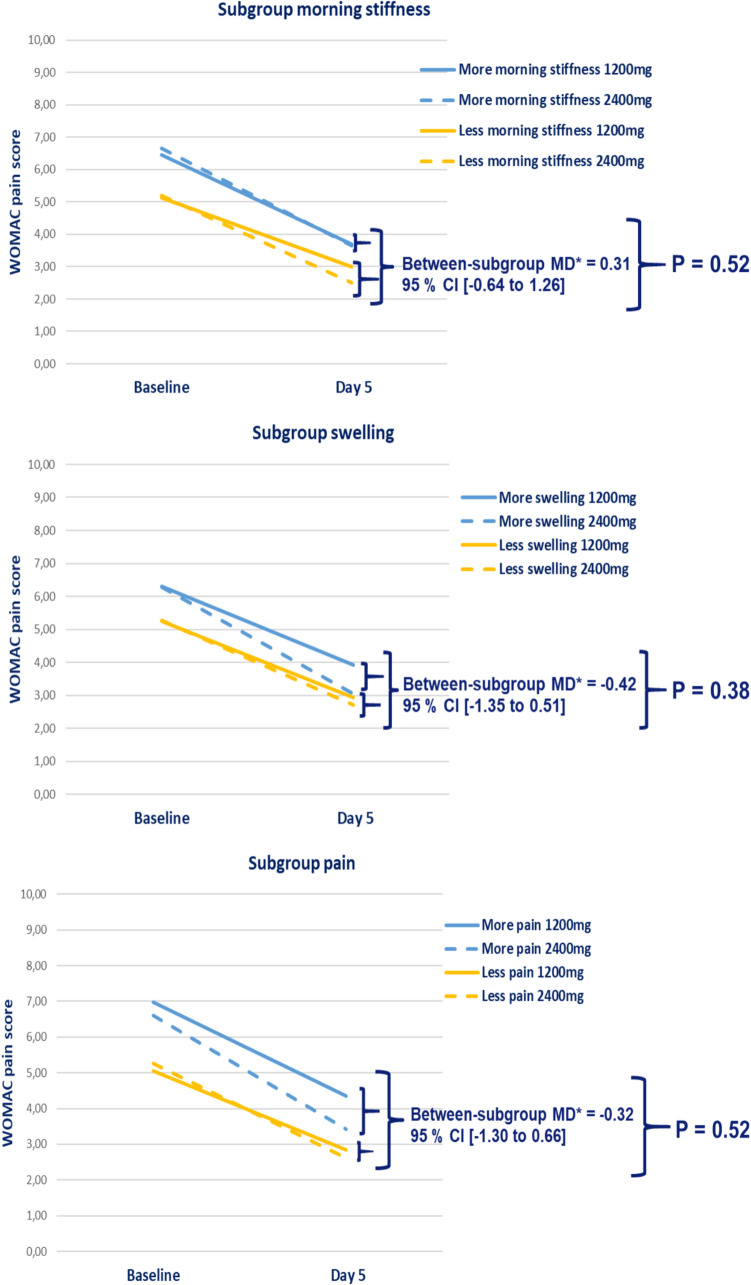
Adjusted mean between-subgroup treatment differences and corresponding significance level of the treatment effect for each subgroup on the secondary outcome measures WOMAC pain score (0–10 range) at day 5 of treatment. *MD = adjusted mean difference; NRS = Numeric Rating Scale; *P* = *p*-value

The adjusted mean between-subgroup treatment differences were all less than 0.32, which is below the clinical relevance margin for all subgroup effects. All values are presented in Table 2, Fig. 3 (WOMAC pain score at day five) and supplementary Fig. 3 (NRS pain score at day three). For completeness, the adjusted mean within-subgroup treatment difference is presented in supplementary Table 2.

### Sensitivity analysis

The full analysis dataset consisted of 122 patients with a reported medical history of knee OA and OA without localization. After excluding five patients due to missing data of the pain severity score at day 5, 117 patients were included in the sensitivity analysis.

**Fig. 4:**
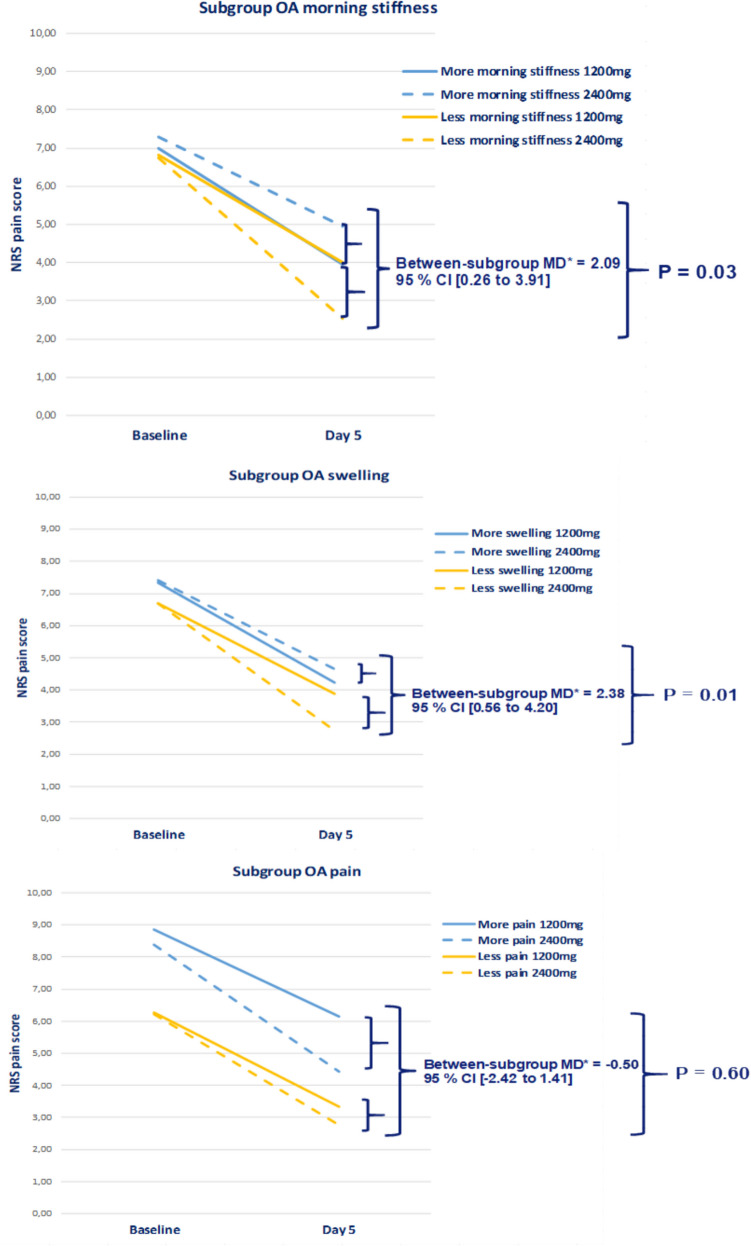
Sensitivity analysis of the interaction between the treatment effect on NRS pain severity (0–10 range) at day 5 and subgroup of patients with osteoarthritis with more and less severe inflammatory symptoms at baseline.*MD = adjusted mean difference; NRS = Numeric Rating Scale; *P* = *p*-value

The sensitivity analysis showed no statistically significant interaction effect on the pain severity at day 5 between the interventions and subgroup of OA patients with more severe knee pain compared to less severe knee pain (*p*-value = 0.6). However, a statistical significant interaction effect on the pain severity at day 5 was seen between the intervention groups and the subgroup of OA patients with less severe morning stiffness (*p*-value = 0.03) and knee swelling (*p*-value = 0.01) (Fig. 4). The adjusted between-subgroup mean treatment differences were 2.09 (95% CI 0.26 to 3.91) and 2.38 (95% CI 0.56–4.20), which is beyond the range of the MCID, indicating clinical relevance.

## Discussion

This secondary analysis evaluated pre-defined subgroups of patients with knee pain flares for the treatment of ibuprofen. Patients with more severe inflammatory symptoms at baseline had no additional benefit of a higher dose of ibuprofen for their pain severity after 5 days (nor day 3) of treatment compared to the subgroups with less severe inflammatory symptoms (*p*-values ≥ 0.28). The adjusted mean between-subgroup treatment differences at both time points were also not clinically relevant, as they were below the clinical relevance margin. Nonetheless, the sensitivity analyses did show a statistically and clinically relevant additional pain reduction with a higher dose of ibuprofen at day 5 of treatment for the subgroups of patients with knee OA with less severe morning stiffness or swelling during a knee flare, compared to the subgroups of patients with knee OA with more severe morning stiffness or swelling.

The absence of a significant interaction effect in the current study might be due to the fact that the etiology and pathogenesis of knee pain flares are unclear, which leads to difficulties in defining subgroups [21]. Although, one of the defined subgroup indicators in this study was knee swelling, which was associated with flares in previous studies [34, 35].

Furthermore, several factors influence the effect modification of a certain subgroup [21]. The study of Atukorala et al. [36] showed a risk prediction model for knee OA flares with the best predictive capacity including higher baseline pain scores as a variable. However, higher baseline pain scores as a subgroup variable did not have an additional benefit from a higher dose oral NSAIDs therapy on pain reduction in this secondary analysis.

The limitations of this study include the following: The original trial data was powered for the overall treatment effect, not for subgroup effects. A priori power analysis was not performed for the current subgroup analysis. As the power was limited to detect statistically significant interactions, the absolute and adjusted mean differences between intervention groups were compared for each subgroup additionally. A post hoc power calculation was not considered useful as many concerns have been raised in literature [37, 38]. A statistical significance could have been detected with more power; however, a clinical relevant finding could still be absent [39]. And the aim of this secondary subgroup analysis was to identify clinically relevant subgroups.

To interpret the results, instead of power analysis, it is recommended to use the 95% confidence intervals (CI) for effect sizes (e.g., mean difference). The range of the 95% CI could also indicate if a clinically meaningful association is possible. For our study results, the range of the 95% CI of the effect estimates is wide, which makes that the estimates of the true effect sizes are not precise. Still, the 95% CI is informative regarding possible false negative findings, as it shows a range of effect sizes that are consistent with the observed data and the true effect size likely lies within this range [37].

Also, the FLARE RCT provided a distinct dataset of primary care patients with knee pain flares. These flares do not only have an inflammation as potential underlying pathology but could have other causes (e.g., clinically undiagnosed gout or avascular necrosis) [40].

As mentioned before, the original FLARE trial intended to investigate the efficacy of a new lipid formulation of ibuprofen, we only included the commonly available soft-gel ibuprofen. The trial did not include a control group, which was not relevant for our research question investigating the between-subgroup differences.

Another limitation is the possibility to apply the original National Institute for Health and Care Excellence (NICE) criteria [41] on the dataset due to insufficient data. The cohort was recruited from the primary care setting. Despite the fact that the selected cohort existed of a younger population, it might represent an early clinical phase OA cohort. Still, a subselection of OA patients was identified as per their reported medical history.

Remarkably, in the current analysis, the higher dose of ibuprofen seems to have more effect in the subgroup of OA patients with less severe morning stiffness and swelling. This could well be an incidental finding, as with the current knowledge, we hypothesized that a higher dose ibuprofen might be more beneficial for the subgroups with more severe inflammatory symptoms at baseline compared to the subgroups with less severe symptoms. In the sensitivity analyses, these subgroups had a high overlap of patients (64%), which might have led to similar result in both subgroups.

Also, NSAIDs had better efficacy on OA-related pain [33], which suggest that flares have mainly inflammation as underlying cause in OA patients [6]. The prevalence of flares in patients with knee OA is 53% in the general practice population, whereas in elderly with knee pain, this ranges from 23 to 32% [42].

It is important to note that an interaction effect between the intervention groups and subgroups with more and less severe inflammatory symptoms might be influenced by the course and duration of a knee pain flare [43]. Literature describes that clinical effects of recommended pharmacological interventions are limited to the first 2–3 weeks of treatment [44, 45]. In a longitudinal study, a median duration of eight days was stated with a relatively quick reduction in pain intensity within 48 h [43]. Our study endpoints were set at days 3 and 5 of the 5-day treatment course. Based on the literature, this would be an expected time frame to find an interaction effect between the intervention groups and subgroups.

Furthermore, the NRS pain score and WOMAC pain subscale scores evaluated the pain over the last 7 and 2 days, respectively. The results of this study had a follow-up of 3 and 5 days. The treatment interaction effect could have been diluted in case pain was improved at day 5 due to the scoring based on the past 7 or 2 days. An accurate result could be obtained when the pain score referred to the same day.

It is also important to mention that the selected population consisted of 94% of white ethnicity, leading to a limitation of this study with an underrepresentation of the other ethnicities (e.g., Asian, Black or African American, Surinamese). Researchers should actively pursue a representative distribution of the ethnicities when recruiting patients for studies in the future.

One of the strengths of this study is the self-reported measures (on NRS and WOMAC scale) of swelling and morning stiffness.

Tanaka et al. [46] demonstrated that a considerable amount of knee OA patients report swelling despite the absence of swelling by objective measurements. Also, Hirata et al. [47] investigated the concordance between clinical assessment and patient-reported assessment of joint synovitis. Their finding showed a superiority of patient self-assessment over the clinical evaluation of joint tenderness.

The results of the primary study supported the rationale for possible subgroup effects [3]. These findings were explorative and could provide additional context to the findings of the trial to tailor individualized treatment opportunities. The current project focused on patient characteristics that are easily obtainable in primary care. Further research is needed with a larger sample size for subgroup analysis of a more specific OA or advanced stage OA population, based on the sensitivity analysis results and the probability of an inflammatory process as underlying pathophysiology of the flare. Taking drug-related adverse events into account, other subgroup indicators, such as polyarthritis, radiographic severity, chronic complaints, or elderly [21], should be investigated as well. Hence, when clinically relevant subgroups of OA patients with knee flares are identified, these results can be readily applied in clinical practice.

### Clinical implication

The clinical implication of a higher ibuprofen dose during a knee pain flare is dependent on patient characteristics as well as the contraindications and adverse effects of the drug [48, 49]. Although the treatment with ibuprofen is for a short duration in the original trial, the adverse effects are dose-dependent [48]. Contraindications for a higher dose of ibuprofen contains having a high gastrointestinal, cardiovascular, or renal risk. Other contraindications might be pharmacological interactions. Patients characteristics, such as age, also influence the risk of the adverse events (11). The primary trial has reported on the GSRS total score as a secondary study endpoint. At baseline of the trial, mean GSRS scores were low, and during the study, only small changes were observed (8). 

## Conclusion

The difference in change in pain severity for patients with knee pain flares treated with 2400 mg versus 1200 mg ibuprofen [3] was independent of the pre-defined subgroups based on more or less severe clinical inflammatory symptoms (morning stiffness, swelling and pain). A higher dose of ibuprofen is therefore not recommended for these specific pre-defined subgroups.

## Supplementary Information

Below is the link to the electronic supplementary material.

ESM 1 (DOCX 552 KB)

## Data Availability

The data are subject to third party restrictions as data are owned by “InFirst Healthcare Ltd.” Disclosures
