# Supplementary material for: Identifying clinically relevant subgroups of patients with knee pain flares for ibuprofen treatment: a secondary analysis
Source: Clin Rheumatol. 2025 Jun 25;44(8):3321–32. doi: 10.1007/s10067-025-07539-0 (PMC12289777; doi:10.1007/s10067-025-07539-0)
Supplement: Supplementary file 1 — (DOCX 552 KB) [file 10067_2025_7539_MOESM1_ESM.docx]

**Supplementary material**

| **Subgroups** | **Severity** | **N** | **Cut-off value NRS score*** | **Prevalence (%)** | **Baseline scores* (mean ± SD)** | **Actual observed effect size^#^** | **Adjusted between-subgroup MD**[95 %CI]** |
| --- | --- | --- | --- | --- | --- | --- | --- |
| Morning stiffness | 1. More | 97 | ≥ 8 | 30.9 | 8.4 ± 0.6 | 0.578 | -0.130 [-1.01 to 0.75]] |
|  | 2. Less | 217 | < 8 | 69.1 | 5.2 ± 1.9 |  | -0.708 [-1.29 to -0.13] |
| Sensation of swelling | 3. More | 103 | ≥ 6 | 30 | 7.0 ± 1.1 | 0.104 | -0.493 [-1.34 to 0.36] |
|  | 4. Less | 211 | < 6 | 70 | 2.2 ± 1.9 |  | -0.597 [-1.19 to -0.002] |
| Pain | 5. More | 84 | ≥ 8 | 26.8 | 8.4 ± 0.6 | -0.502 | -0.931 [-1.9 to 0.01] |
|  | 6. Less | 230 | < 8 | 73.2 | 6.2 ± 0.8 |  | -0.428 [-0.99 to 0.13] |

***Supplementary table 1.*** *Subgroup definitions and baseline scores per subgroup*

*N = number of patients (randomized) per group; NRS = Numeric Rating Scale; SD = standard deviation;* *MD = adjusted mean difference; CI = confidence interval*

**NRS scores (stiffness, swelling, and pain) at baseline on a 0-10 scale range.*

*#For the primary outcome pain severity at day 5*

***Adjusted for treatment center and baseline score of the outcome. Between-subgroup mean treatment difference is the mean difference between the subgroup with less and more severe complaints for the treatment of 2400 mg versus 1200 mg ibuprofen treatment.*

|  | **Subgroup with less severe complaints** | | **Subgroup with more severe complaints** | |
| --- | --- | --- | --- | --- |
|  | **Treatment effect MD*** | **95% CI** | **Treatment effect MD*** | **95% CI** |
| **Morning stiffness** | | | | |
| Pain severity day 5 | 0.708 | 0.129 to 1.288 | 0.130 | -0.749 to 1.009 |
| WOMAC pain day 5 | -0.534 | -1.054 to 0.014 | -0.222 | -1.010 to 0.565 |
| Pain severity day 3 | -0.402 | -0.912 to 0.108 | -0.088 | -0.859 to 0.684 |
| **Swelling** | | | | |
| Pain severity day 5 | 0.597 | 0.002 to 1.193 | 0.493 | -0.356 to 1.343 |
| WOMAC pain day 5 | -0.310 | -0.221 to 0.840 | -0.729 | -1.486 to 0.28 |
| Pain severity day 3 | -0.376 | -0.898 to 0.147 | -0.254 | -1.003 to 0.495 |
| **Pain** | | | | |
| Pain severity day 5 | 0.428 | -0.130 to 0.987 | 0.931 | -0.006 to 1.868 |
| WOMAC pain day 5 | -0.364 | -0.865 to 0.137 | -0.682 | -1.522 to 0.158 |
| Pain severity day 3 | -0.236 | -0.729 to 0. 257 | -0.602 | -1.427 to -0.223 |

***Supplementary table 2.*** *Subgroup analyses. Subgroup analyses (interaction intervention x subgroup and adjusted* mean differences) for pain severity at day 5 (primary outcome), WOMAC pain at day 5 (secondary outcome) and pain severity at day 3 (secondary outcome) for N =308. *Adjusted for treatment center and baseline score of the outcome. Treatment effect is the difference between 2400 mg and 1200mg within each subgroup (adjusted mean within-subgroup treatment difference). CI = confidence interval; MD = adjusted mean difference.*

***Supplementary figure 3.*** *Adjusted mean between-subgroup treatment differences and corresponding significance level of the treatment effect for each subgroup on the secondary outcome measures.*


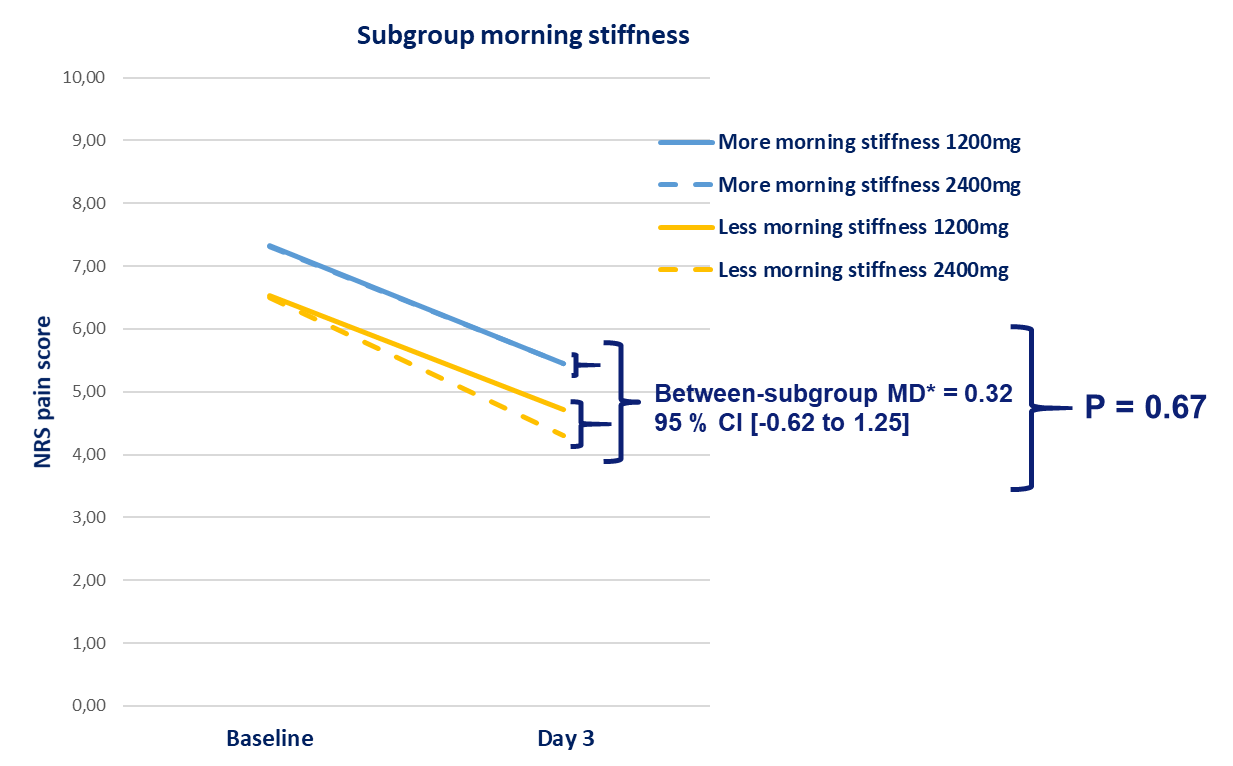

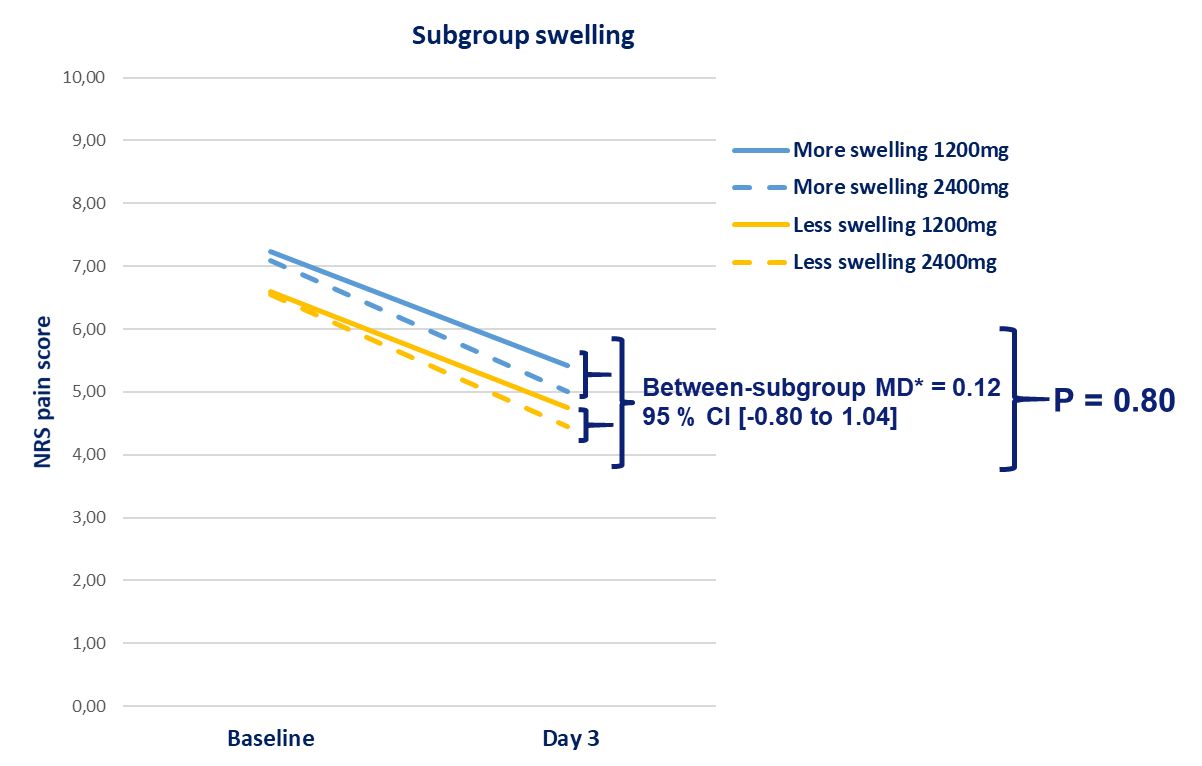

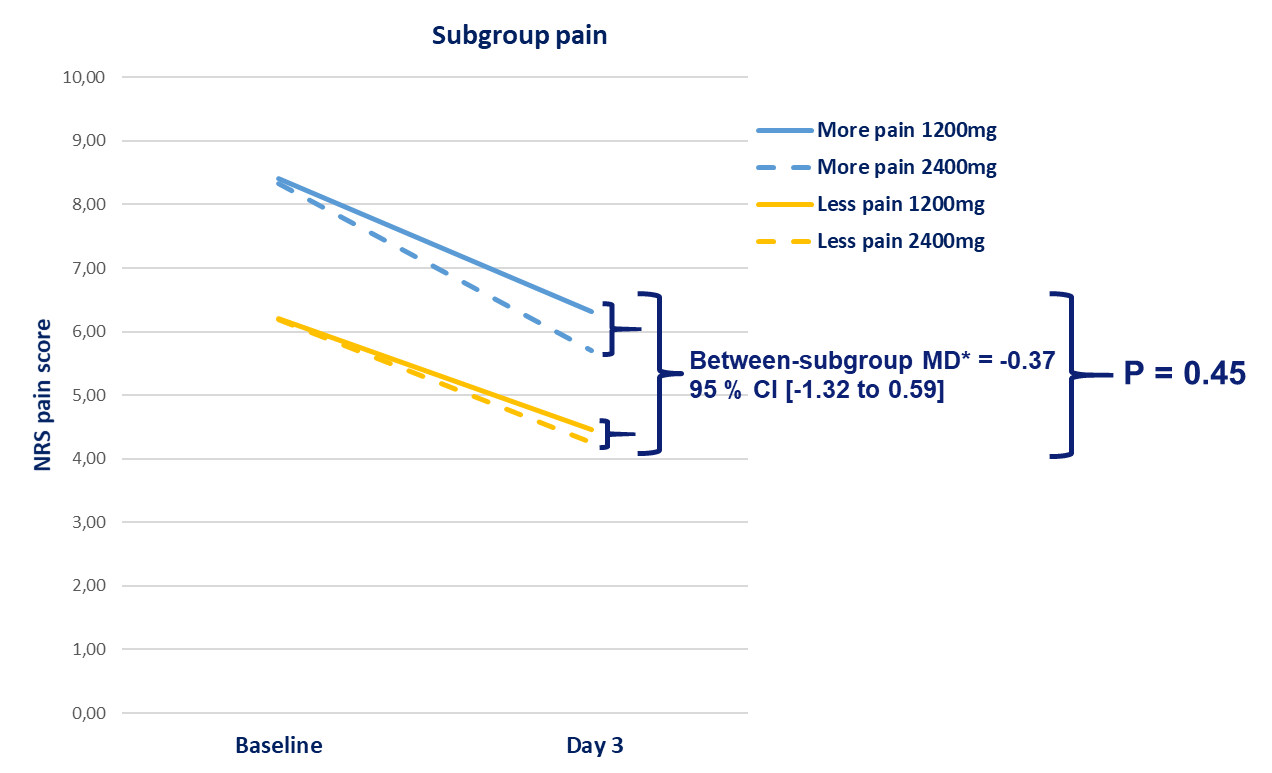


*NRS pain severity score (0-10 range) at day 3 of treatment. * MD = adjusted mean difference; NRS = Numeric Rating Scale; P = p-value*
